# Immune diversity sheds light on missing variation in worldwide genetic diversity panels

**DOI:** 10.1371/journal.pone.0206512

**Published:** 2018-10-26

**Authors:** Laurent Abi-Rached, Philippe Gouret, Jung-Hua Yeh, Julie Di Cristofaro, Pierre Pontarotti, Christophe Picard, Julien Paganini

**Affiliations:** 1 Aix Marseille Univ, IRD, APHM, MEPHI, IHU-Méditerranée Infection, Marseille, France; 2 CNRS, Marseille, France; 3 XEGEN Company, Gemenos, France; 4 Etablissement Français du Sang PACA Corse, Biologie des Groupes Sanguins, Marseille, France; 5 Aix Marseille Univ, CNRS, EFS, ADES, "Biologie des Groupes Sanguins", Marseille, France; University of Utah, UNITED STATES

## Abstract

Defining worldwide human genetic variation is a critical step to reveal how genome plasticity contributes to disease. Yet, there is currently no metric to assess the representativeness and completeness of current and widely used data on genetic variation. We show here that Human Leukocyte Antigen (HLA) genes can serve as such metric as they are both the most polymorphic and the most studied genetic system. As a test case, we investigated the 1,000 Genomes Project panel. Using high-accuracy *in silico* HLA typing, we find that over 20% of the common HLA variants and over 70% of the rare HLA variants are missing in this reference panel for worldwide genetic variation, due to undersampling and incomplete geographical coverage, in particular in Oceania and West Asia. Because common and rare variants both contribute to disease, this study thus illustrates how HLA diversity can detect and help fix incomplete sampling and hence accelerate efforts to draw a comprehensive overview of the genetic variation that is relevant to health and disease.

## Introduction

**Fig 1:**
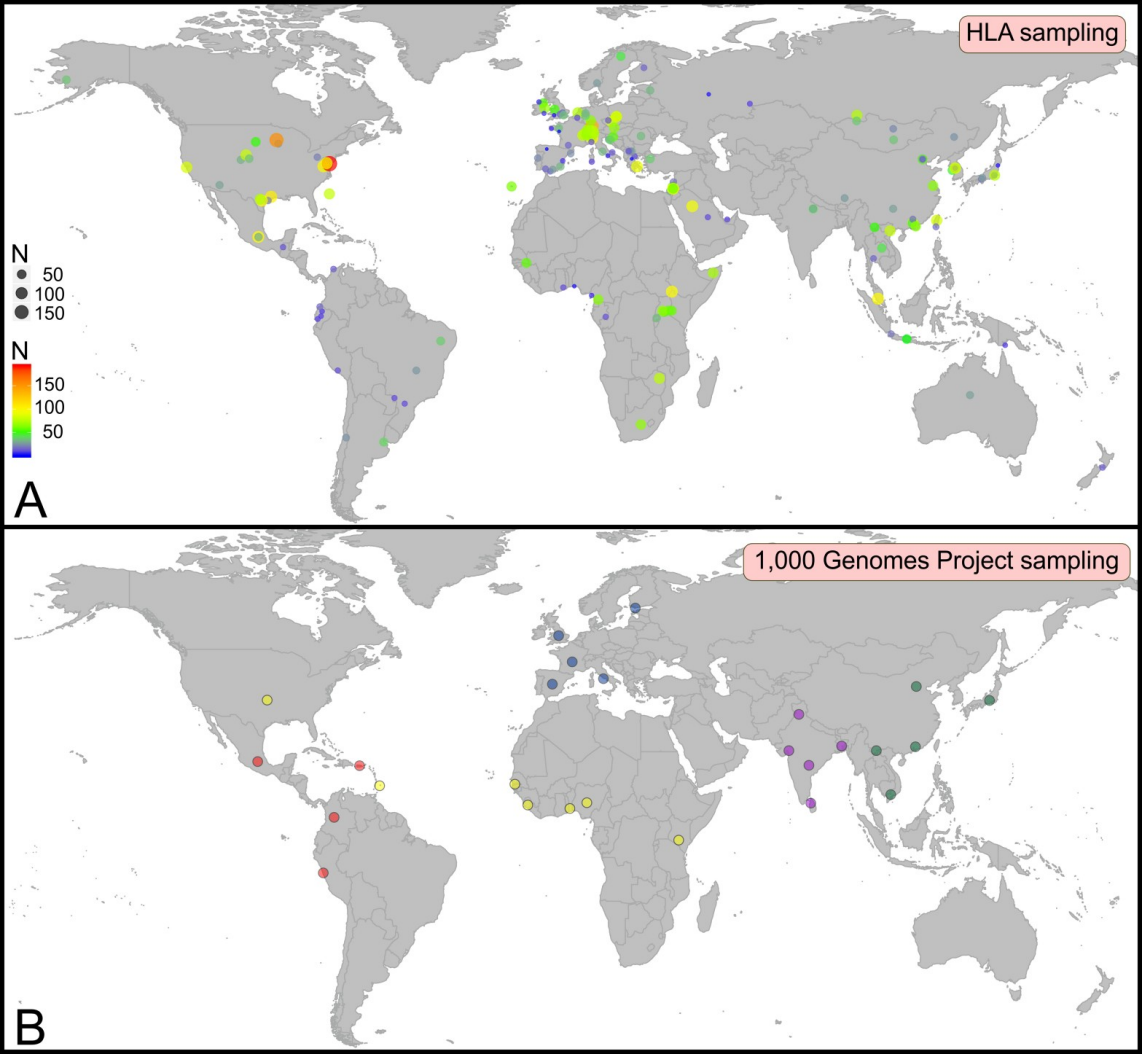
HLA sampling (A) is much more widespread than that of the 1,000 Genomes Project (B), which represents the current reference panel of human genomewide diversity. A. Circles represent the populations that were HLA typed. Colors and size of the circles correspond to the number of common alleles observed in each population (scales in the bottom left corner). B. Circles represent the region of origin for each of the 26 populations of the 1,000 Genomes Project. Each population has a color that corresponds to the associated geographical region: Europe (blue), Africa (yellow), Americas (red), South Asia (purple), and East Asia (green). A-B. Maps were generated using the ggplot2 package in R [16] and the world database.

Defining worldwide human genetic variation at a genomewide level promises breakthroughs to reconstruct human evolution [1] and to define how genetic variation contributes to disease, hence paving the way towards precision medicine [2]. By decreasing sequencing costs by orders of magnitude [3], Next-Generation sequencing (NGS) facilitated the development of large scale genome sequencing projects [4–6] aimed at fulfilling this goal. Notably, the 1,000 Genomes Project is currently the reference panel for human genetic diversity [7], having targeted 2,693 individuals from 26 worldwide populations (S1 Fig), with the aim to define common genetic variants whose frequency is higher than 1% in populations. Although completion of the 1,000 Genomes Project represents a milestone for human genomics, there is currently no metric to assess how representative such reference panels for genomewide variation are and sampling bias in particular remains a concern [8]. Here we propose to use Human Leukocyte Antigen (HLA) diversity as a benchmark to perform this assessment. Indeed, because of their critical role in immunity and reproduction, HLA genes evolved to become the most polymorphic human genes, with over 11,000 distinct protein variants encoded by the five most variable *HLA* loci (*HLA-A*, *-B*, *-C*, *-DRB1* and *-DQB1*) [9], hence making them superb markers of human diversity [10]. Their impact on health and disease [11] and in transplantation medicine [12–14] in particular also ensured that HLA sampling is second to none, with hundreds of populations and millions of individuals studied worldwide [15]. The HLA system is thus both the most polymorphic and the most studied genetic system (Fig 1A), and HLA sampling is second to none and considerably more widespread than that of the 1,000 Genomes Project panel for example (Fig 1B). HLA variation can therefore provide a high resolution picture of the representativeness of reference panels for genomewide diversity [15].

## Materials and methods

### 1,000 Genomes Project sequence data

Exome sequence data for all 2,693 individuals forming the 1,000 Genomes Project was obtained (S1 Fig) and then spilt into two groups: a first set of 992 exomes for which there was already available HLA typing data [17] and a second set of 1,701 exomes for which there was no HLA typing data. HLA typing was then performed with the Polymorphisms to Phenotypes for Medicine (PolyPheMe) software, an *in silico* solution to perform highly-accurate HLA typing from any type of NGS data (see S1 File). In this study, the first set of data was used to evaluate PolyPheMe’s performance and the second set was used for *de novo*, *in silico* HLA typing.

### PolyPheMe HLA typing tool

The PolyPheMe software (Xegen, France) can perform high accuracy HLA typing for five genes (*HLA-A*, *-B*, *-C*, *-DRB1*, and *-DQB1*) for a wide range of sequence data, including whole exome or whole genome data (see S1 File for details about PolyPheMe). All analyses were performed with PolyPheMe v1.2 on exome sequences using the IMGT 3.28 database [9] as reference.

### Evaluation of PolyPheMe performance

To investigate the performance of PolyPheMe, we first analyzed the subset of the 1,000 Genomes Project for which there was already available HLA typing data [17]. *In silico* typing was thus performed for the *HLA-A*, *-B*, *-C*, *-DRB1*, and *-DQB1* genes for 992 individuals. 294 individuals were excluded for the *HLA-DQB1* analysis due to lack of data likely linked to enrichment issues and a total of 9,332 types were generated.

The types obtained with PolyPheMe were then compared to those already established [17], and three categories were defined: 1/‘concordant’ when the result with PolyPheMe is precise (only one type isolated) and compatible with previously established types, 2/‘imprecise’ when the result with PolyPheMe includes multiple possible types but at least one of them is contained in the list of previously established types, and 3/‘discordant’ when the results are not compatible (S2 Fig).

Results initially labelled as‘imprecise’ or‘discordant’ were further investigated through manual inspection of the sequence reads. For 21 individuals, genomic DNA was also obtained from Coriell Institute and a new HLA typing was performed with a commercial kit (Holotype, Omixon) using NGS. Results were directly analyzed both with commercial Omixon software and PolyPheMe software. This verification step confirmed that 105 typing results initially identified as‘discordant’ and four typing results initially identified as imprecise were in fact correctly typed by PolyPheMe (S3 Fig).

After verification of the typing results, the performance of PolyPheMe reaches a precision of 99.7% (9,300/9,332) and only 0.3% of the types are erroneous (32/9,332) (S2 Fig). Analysis of these 32 cases shows that 29 of them represent unique cases and all 29 of these unique variants were observed in the panel in other individuals (S1 and S2 Tables). This shows that these errors are not biased towards particular variants. Instead, analysis of these cases indicate that the problem stems from under or overrepresentation of one of the two alleles of the locus.

After validating the performance of PolyPheMe, the complete panel of the 1,000 Genome Project was investigated (S4 Fig).

### Defining common HLA variants

For each of the five target HLA genes, we established a list of the variants having an allele frequency >1% in at least one population in the Allele Frequency Net Database [15]. Populations with a low sample size can produce incorrect estimates of the real allele frequencies and, conversely, setting too high a limit for the minimum population size can eliminate populations and lead to a lack of coverage for certain geographical regions. Thus a threshold must be selected to reduce the noise produced by populations with low sampling and to maximize geographical coverage. Using this approach, we selected a minimum population size of 150 individuals (S5 Fig) and 340 distinct alleles were defined as common (S3 Table). As an alternative, we also performed an analysis where the number of times an allele was observed in populations is used as a measure of how common it is (S6 Fig).

### Definition of the common HLA haplotypes

Common five-genes haploypes (*HLA-A*, *-B*, *-C*, *-DRB1*, and *-DQB1*) were defined as those having a frequency >1% in at least one population in the National Bone Marrow Program reference panel [18] (S7 Fig), the largest source of HLA haplotype data, with a total of 74,425 different haplotypes already observed in one or more population.

### *HLA* haplotype prediction for the 1,000 Genomes Project panel

The PolyPheMe software integrates a module for haplotype prediction based on a previously described methodology [19]. Briefly, using the precise, five-locus HLA genotype, all haplotype combinations are first generated (S4 Table). Then, in selecting the most likely haplotype structures, a priority is given to combinations maximizing the frequency observed in a single population. If a genotype cannot be explained by a haplotype combination in a unique population, the combination that maximizes frequency across all populations is selected. If no haplotype combination can explain the observed genotype, the haplotype with the best frequency is selected and the other haplotype is predicted.

## Results

As a first step to compare the HLA diversity observed in the 1,000 Genomes Project to that commonly observed in worldwide populations, we set to define the HLA types of all 2,693 individuals forming the 1,000 Genomes Project panel (S1 Fig) at the five most polymorphic HLA loci: *HLA-A*, *-B*, *-C*, *-DRB1* and *-DQB1*. This analysis used the Polymorphisms to Phenotypes for Medicine (PolyPheMe) software, an *in silico* solution to perform highly-accurate HLA Typing from any type of NGS data (see Methods and S1 File). As an initial test, PolyPheMe’s performance was first assessed on the 992 individuals of the panel that had already been HLA typed using standard methodology [17]. This comparison revealed a 98.5% concordance between PolyPheMe’s results and previous HLA typing (S2 Fig), but after careful sequence inspection and targeted resequencing (S3 Fig), the accuracy was estimated to be higher, reaching 99.7% (S2 Fig, S1 Table). Following this validation step, PolyPheMe was then used to determine the HLA types for the remaining 1,701 individuals for whom there was no prior information (S4 Fig, S2 Table). Out of the 26,342 cases of *in silico* typing performed, only 132 types (0.5%) were not precisely resolved (i.e. defined without ambiguities at a two-field level) and for an additional 32 cases (0.1%), no alleles were identified (S4 Fig). Thus 26,178 precise types (99.4%) were obtained and represent 414 distinct variants: 84 for *HLA-A*, 160 for *HLA-B*, 67 for *HLA-C*, 31 for *HLA-DQB1*, and 72 for *HLA-DRB1*.

**Fig 2:**
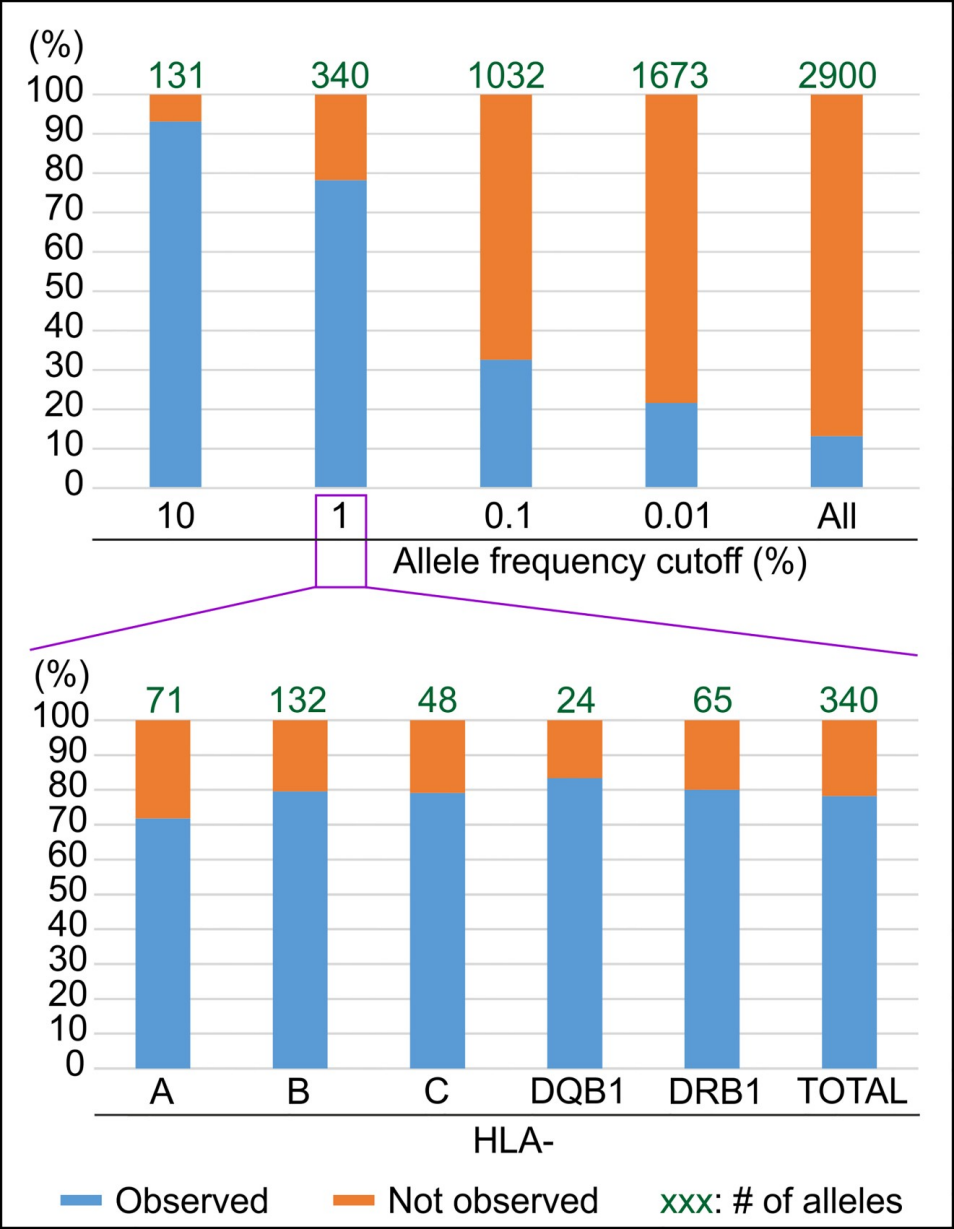
The HLA diversity in the 1000 Genomes Project panel only represents 78% of the expected diversity for the alleles with a frequency >1%. The top part of the figure shows the % match (Y axis) between the expected HLA diversity at different frequency cutoffs (X axis) and the HLA diversity observed in the 1,000 Genomes Project panel. For each frequency cutoff, the number of expected alleles is displayed at the top of each histogram. The bottom part of the figure displays the same information on a locus by locus basis for the 1% cutoff.

To assess how well the variants observed in the 1,000 Genomes Project panel represent common HLA variation, we used data from the Allele Frequency Net Database to define a common set of variants corresponding to those whose frequency is higher than 1% in populations with sufficient sampling (n>150 individuals, see Methods and S5 Fig). From the analysis of 256 such populations, 340 HLA variants were observed (S3 Table): comparison of these 340 variants to the 414 variants observed in the 1,000 Genomes Project panel shows that the two sets only share 266 variants in common (Fig 2). Thus 74 of the 340 common HLA variants (22%) are missing in the 1,000 Genomes Project panel. That the missing fraction is homogenously distributed across the five HLA genes investigated, ranging between 17 and 28%, shows that this number is not due to a sampling issue with a given gene but is rather a general representation of the common HLA variation missing in the 1,000 Genomes Project panel.

**Fig 3:**
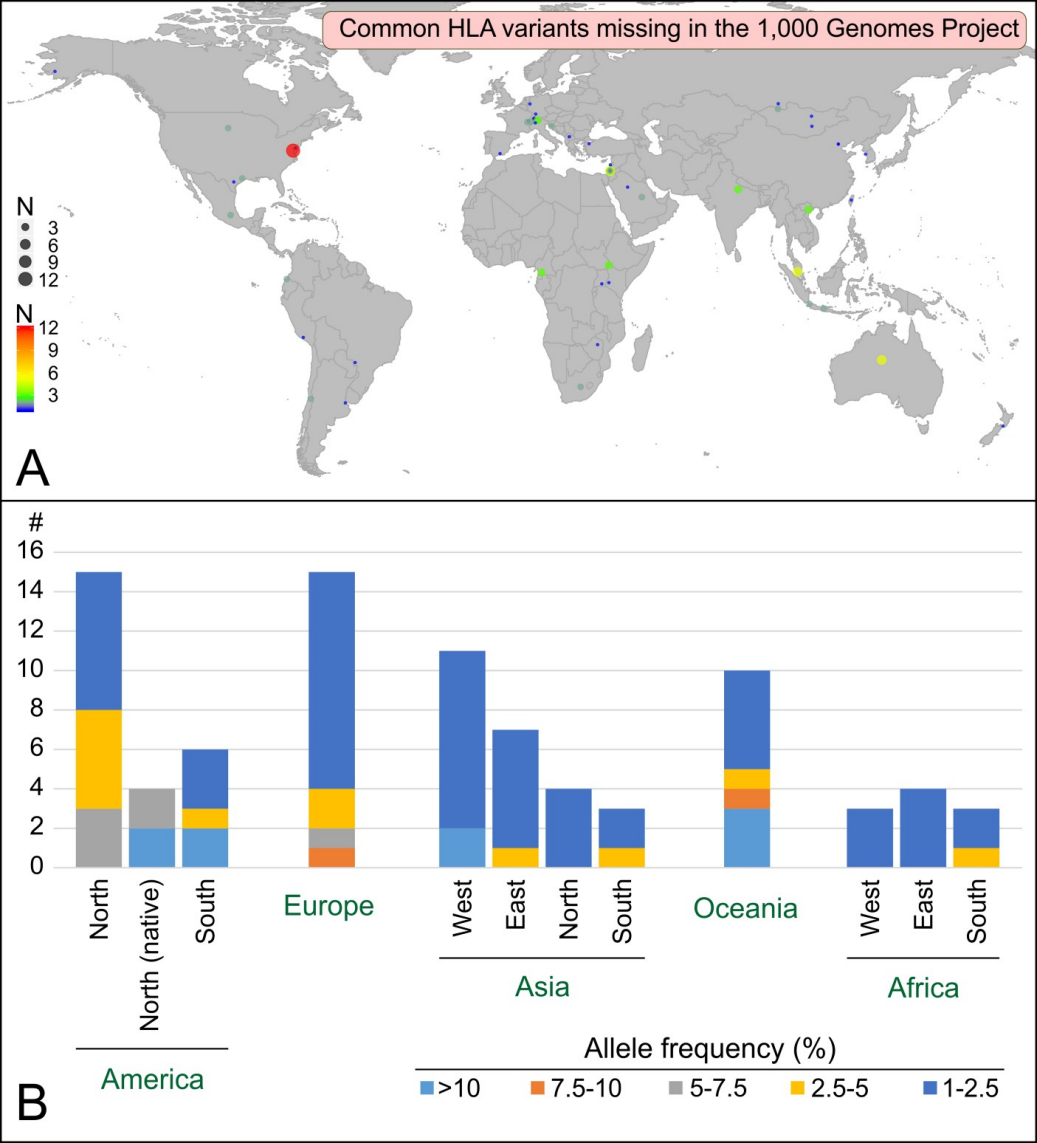
The common HLA alleles that are missing in the 1,000 Genome Project panel define a worldwide distribution with variable frequencies that range from low (1–2.5%) to high (>10%). A. Worldwide distribution of the populations harboring alleles that are missing in the 1,000 Genomes Project. Colors and size of the circles correspond to the number of common alleles observed in each population (scale on the bottom left corner). The map was generated using the ggplot2 package in R [16] and the world database. B. Geographical distribution of the alleles that are missing in the 1,000 Genomes Project. For each region, the number of distinct alleles is given together with their maximum frequencies.

To understand why 74 common HLA variants are not present in the 1,000 Genomes Project panel, we investigated their geographical and population distribution (Fig 3A). This analysis illustrates two limitations of the 1,000 Genomes Project sampling: missing populations and undersampled regions. The former refers to populations in regions of the world that are not represented in the 1,000 Genomes Project sampling. Indeed, ten and eleven of the missing variants originate for example from populations in Oceania and West Asia, respectively, two geographical regions that are not represented in the 1,000 Genomes Project sampling. Importantly, in those regions, the missing variants can be extremely common, with frequencies higher than 10% (Fig 3B). The latter, ‘undersampled regions’, corresponds to regions where the sampling does not fully grasp the population diversity of the region. This category includes both a lack of depth in the sampling in terms of the number of individuals studied and an insufficient geographical sampling within a region. For example, while five European populations are included in the 1,000 Genomes Project, fifteen common variants were missed: ten whose frequency is 1–2.5% and that might have been characterized with more sampling depth, and four whose frequency is >2.5% (2.5–10%) that were likely missed due to the limited geographical sampling within the region.

Thus, while a large fraction (78%) of the common HLA alleles are observed in the 1,000 Genomes Project panel, the HLA benchmark underlines two important areas of improvement. First, a significant fraction (7%) of the very common variants (frequency >10%) are missing in the 1,000 Genomes Project panel. Second, the representativeness of the panel drops dramatically when less common HLA variants are considered: to 33% for example if a larger set of expected alleles with frequency >0.1% is considered (Fig 2). A similar result is obtained if, instead of using a fixed frequency cutoff, the reference set of expected variants is set to include all the variants observed at least five times in populations, as only ~29% of them are observed in the 1,000 Genomes Project panel (S6 Fig). Likewise, assessing the 1,000 Genomes Project in terms of HLA haplotype diversity, a relevant unit for disease association studies, shows that only 37 of the 58 haplotypes (63.8%) whose frequency is >1% in the largest HLA haplotype database (National bone marrow program database [18]) are observed in the 1,000 Genomes Project panel (S7 Fig). That 20 of the 21 missing haplotypes are associated with native populations from the USA or from Oceanian populations, two groups already identified as ‘missing’ (Fig 3), shows that ‘missing populations’ are also the main source of missing haplotypes.

## Discussion

Worldwide HLA diversity is thus a helpful metric to assess the representativeness of panels of human genetic diversity, as it can define both the common and rare genetic variation that is missing, with both types of variation having potential to impact expression and contribute to individual disease risk [20–22]. The approach isolated coverage issues in a reference panel as widely used as the 1,000 Genomes Project panel. While some of the results are intuitive, like the missing variation linked to regions of the world that were not sampled, HLA variation can help assess how much is missing and isolate missing information that is more difficult to predict, like undersampling in particular geographical regions. This information can in turn be used to improve existing panels and assess how relevant are new panels, a critical step to produce an exhaustive catalogue of human variation [23] and develop new therapeutic solutions [24]. This is important because while genomics is a critical step to understand human diversity [25], it is still failing due to historical bias in sampling that focused too much on individuals of European ancestry [8]. The 1,000 Genomes Project was the first to attempt to provide genomewide data on global human diversity but complementary projects that attempt to increase sample size [26] or focus on specific populations [6, 27] are also developing, together with approaches that gather and compile data from multiple sources [28]. Yet all these approaches to generate a better overview of human genetic variation require a metric to assess how successful they are and we show here that HLA variation is a compelling system to perform that assessment.

## Supporting information

S1 Fig 1,000 Genomes Project sampling. The 1,000 Genomes Project sampling is given on a regional basis (top part) or on a population basis (bottom part). In both cases the number of individuals used to evaluate PolyPheMe’s performance is also given (performance test column).
(PDF)

S2 Fig PolyPheMe’s performance before and after the verification steps. PolyPheMe’s performance was assessed on a subset of 992 individuals of the 1,000 Genomes Project panel who were already HLA typed using standard methodology. This figure summarizes the concordant/correct, imprecise, and discordant/erroneous results obtained before and after the validation step (S3 Fig).
(PDF)

S3 Fig Validation of PolyPheMe results. PolyPheMe’s performance was assessed on a subset of 992 individuals of the 1,000 Genomes Project panel who were already HLA typed using standard methodology. 192 HLA types out of 9,332 were initially characterized either as imprecise or discordant comparing to existing results (S2 Fig). For 109 of these 192 cases, we could show that the PolyPheMe results were correct: this figure summarizes the validation steps used for those 109 cases.
(PDF)

S4 Fig In silico HLA typing of the 1,000 Genomes Project panel by PolyPheMe. This figure summarizes the total number of in silico HLA types realized by PolyPheMe on a locus-by-locus basis. For each locus, the number of precise and imprecise types is given, together with the number of cases for which no type was obtained.
(PDF)

S5 Fig Impact of the population size threshold (horizontal axis) on the number of populations used (vertical axis on the left, blue line), on the number of common HLA variants defined (vertical axis on the left, orange line), and on the fraction of common HLA variants observed in the 1,000 Genomes Project panel (vertical axis on the right, grey line). A threshold of at least 150 individuals per population was used for the final analysis (dotted line).
(PDF)

S6 Fig More than 70% of the HLA variants observed at least five times in populations are missing in the 1,000 Genomes Project panel. For each occurrence threshold (minimum number of observations of a given allele in populations), the number of alleles of *HLA-A*, *-B*, *-C*,–*DQB1*, and–*DRB1* observed with this minimum occurrence is given (line ‘1.’). The second line (‘2.’) indicates how many of the alleles listed in 1. are also observed in the 1,000 Genomes Project panel. Finally the third line corresponds to the fraction ‘2./1’. For example, 1,202 *HLA-A*, *-B*, *-C*,–*DQB1*, and–*DRB1* alleles are observed at least five times in populations. Out of those 1,202 alleles, 354 are also observed in the 1,000 Genomes Project panel, which corresponds to a fraction of 0.29.
(PDF)

S7 Fig Common HLA haplotypes. This figure summarizes the common HLA haplotypes and the populations that harbor them with frequency > 1%. The third column gives the number of observations of the haplotypes in the 1,000 Genomes Project panel. Population codes correspond to those described in S8 Fig. Two haplotypes that are not observed in the 1,000 Genomes Project are in bold because they carry common HLA variants (HLA-C*08:*06* and HLA-DRB1*14:*08*).
(PDF)

S8 Fig Population codes for the haplotype analysis. This figure provides the population codes used in the haplotype analysis (S7 Fig).
(PDF)

S1 Table Evaluation of PolyPheMe performance. This file provides the results of the analysis with PolyPheMe for the subset of the 1,000 Genomes Project for which there was already available HLA typing data. For 928 of the 992 individuals, the PolyPheMe results were ‘correct’ for all loci (first tab ‘Correct typing’). In some cases (marked by asterisks), the PolyPheMe typing was validated through manual verifications (see Methods). For 64 of the 992 individuals (tab ‘Others’), at least one HLA type was either imprecise (marked in orange) or erroneous (marked in red). The correct type was obtained through manual verifications (see Methods) and is given in green below the imprecise or erroneous type.
(XLSX)

S2 Table Final HLA types for the complete 1,000 Genome Project panel. This table gives the final HLA types in our analysis for the 2,693 donors of the 1,000 Genome Project panel.
(XLSX)

S3 Table Common *HLA* variants. This Table lists the 340 distinct HLA variants defined as ‘common’, i.e. whose frequency is higher than 1% in populations with sufficient sampling (see Methods).
(XLSX)

S4 Table Five-locus *HLA* haplotypes predicted for the donors of the 1,000 Genome Project panel. This Table provides the predicted five-locus *HLA* haplotypes (*HLA-A*, *-B*, *-C*, *-DRB1*, *-DQB1*) for the donors of the 1,000 Genome Project. For each donor, the two haplotypes are given, together with the population where each was observed with the highest frequency (population codes are given in S8 Fig).
(XLSX)

S1 File PolyPheMe HLA typing tool. A description of PolyPheMe software is provided: general description, typing strategy, typing steps, results, and computing power requirements and availability.
(PDF)

## References

[1] Nielsen R, Akey JM, Jakobsson M, Pritchard JK, Tishkoff S, Willerslev E. Tracing the peopling of the world through genomics. Nature. 2017;541(7637):302–10. 10.1038/nature21347 . 28102248 PMC5772775

[2] Aronson SJ, Rehm HL. Building the foundation for genomics in precision medicine. Nature. 2015;526(7573):336–42. 10.1038/nature15816 26469044 PMC5669797

[3] Wetterstrand K. DNA sequencing costs: data from the NHGRI Genome Sequencing Program (GSP). http://wwwgenomegov/sequencingcostsdata. 2017.

[4] Pagani L, Lawson DJ, Jagoda E, Morseburg A, Eriksson A, Mitt M, et al Genomic analyses inform on migration events during the peopling of Eurasia. Nature. 2016;538(7624):238–42. 10.1038/nature19792 27654910 PMC5164938

[5] Telenti A, Pierce LC, Biggs WH, di Iulio J, Wong EH, Fabani MM, et al Deep sequencing of 10,000 human genomes. Proceedings of the National Academy of Sciences of the United States of America. 2016;113(42):11901–6. 10.1073/pnas.1613365113 27702888 PMC5081584

[6] Mallick S, Li H, Lipson M, Mathieson I, Gymrek M, Racimo F, et al The Simons Genome Diversity Project: 300 genomes from 142 diverse populations. Nature. 2016;538(7624):201–6. 10.1038/nature18964 27654912 PMC5161557

[7] Genomes Project Consortium, Auton A, Brooks LD, Durbin RM, Garrison EP, Kang HM, et al A global reference for human genetic variation. Nature. 2015;526(7571):68–74. 10.1038/nature15393 26432245 PMC4750478

[8] Popejoy AB, Fullerton SM. Genomics is failing on diversity. Nature. 2016;538(7624):161–4. 10.1038/538161a 27734877 PMC5089703

[9] Robinson J, Halliwell JA, Hayhurst JD, Flicek P, Parham P, Marsh SG. The IPD and IMGT/HLA database: allele variant databases. Nucleic acids research. 2015;43(Database issue):D423–31. 10.1093/nar/gku1161 25414341 PMC4383959

[10] Parham P. MHC class I molecules and KIRs in human history, health and survival. Nat Rev Immunol. 2005;5(3):201–14. 10.1038/nri1570 . 15719024

[11] Dendrou CA, Petersen J, Rossjohn J, Fugger L. HLA variation and disease. Nat Rev Immunol. 2018 10.1038/nri.2017.143 . 29292391

[12] Flomenberg N, Baxter-Lowe LA, Confer D, Fernandez-Vina M, Filipovich A, Horowitz M, et al Impact of HLA class I and class II high-resolution matching on outcomes of unrelated donor bone marrow transplantation: HLA-C mismatching is associated with a strong adverse effect on transplantation outcome. Blood. 2004;104(7):1923–30. 10.1182/blood-2004-03-0803 . 15191952

[13] Ren EC. The role of HLA matching in unrelated donor bone marrow transplant. Transplantation proceedings. 2000;32(7):1541–2. . 11119825 10.1016/s0041-1345(00)01319-1

[14] Ruggeri L, Capanni M, Casucci M, Volpi I, Tosti A, Perruccio K, et al Role of natural killer cell alloreactivity in HLA-mismatched hematopoietic stem cell transplantation. Blood. 1999;94(1):333–9. . 10381530

[15] Gonzalez-Galarza FF, Christmas S, Middleton D, Jones AR. Allele frequency net: a database and online repository for immune gene frequencies in worldwide populations. Nucleic acids research. 2011;39(Database issue):D913–9. 10.1093/nar/gkq1128 21062830 PMC3013710

[16] Wickham H. ggplot2: Elegant Graphics for Data Analysis: Springer-Verlag New York; 2016.

[17] Gourraud PA, Khankhanian P, Cereb N, Yang SY, Feolo M, Maiers M, et al HLA diversity in the 1000 genomes dataset. PloS one. 2014;9(7):e97282 10.1371/journal.pone.0097282 24988075 PMC4079705

[18] Gragert L, Madbouly A, Freeman J, Maiers M. Six-locus high resolution HLA haplotype frequencies derived from mixed-resolution DNA typing for the entire US donor registry. Human immunology. 2013;74(10):1313–20. 10.1016/j.humimm.2013.06.025 . 23806270

[19] Magalon J, Billard-Daufresne LM, Gilbertas C, Hermanche E, Simon S, Lemarie C, et al Assessing the HLA diversity of cord blood units collected from a birth clinic caring for pregnant women in an ethnically diverse metropolitan area. Transfusion. 2014;54(4):1046–54. 10.1111/trf.12379 . 23944705

[20] Boycott KM, Vanstone MR, Bulman DE, MacKenzie AE. Rare-disease genetics in the era of next-generation sequencing: discovery to translation. Nature reviews Genetics. 2013;14(10):681–91. 10.1038/nrg3555 . 23999272

[21] Long T, Hicks M, Yu HC, Biggs WH, Kirkness EF, Menni C, et al Whole-genome sequencing identifies common-to-rare variants associated with human blood metabolites. Nature genetics. 2017;49(4):568–78. 10.1038/ng.3809 . 28263315

[22] Li X, Kim Y, Tsang EK, Davis JR, Damani FN, Chiang C, et al The impact of rare variation on gene expression across tissues. Nature. 2017;550(7675):239–43. 10.1038/nature24267 . 29022581 PMC5877409

[23] Stenson PD, Mort M, Ball EV, Evans K, Hayden M, Heywood S, et al The Human Gene Mutation Database: towards a comprehensive repository of inherited mutation data for medical research, genetic diagnosis and next-generation sequencing studies. Human genetics. 2017;136(6):665–77. 10.1007/s00439-017-1779-6 28349240 PMC5429360

[24] Scott DA, Zhang F. Implications of human genetic variation in CRISPR-based therapeutic genome editing. Nature medicine. 2017;23(9):1095–101. 10.1038/nm.4377 . 28759051 PMC5749234

[25] Goldstein DB, Cavalleri GL. Genomics: understanding human diversity. Nature. 2005;437(7063):1241–2. 16251937 10.1038/4371241a

[26] Caulfield C, Davies D, Dennys D, Elbahy E, Fowler F, Hill H, et al The 100,000 Genomes Project Protocol. 2017 10.6084/m9.figshare.4530893.v3

[27] Choudhury A, Ramsay M, Hazelhurst S, Aron S, Bardien S, Botha G, et al Whole-genome sequencing for an enhanced understanding of genetic variation among South Africans. Nature communications. 2017;8(1):2062 10.1038/s41467-017-00663-9 . 29233967 PMC5727231

[28] Bahcall OG. Genetic variation: ExAC boosts clinical variant interpretation in rare diseases. Nature reviews Genetics. 2016;17(10):584. 10.1038/nrg.2016.121 27629930

