# Supplementary material for: Immune diversity sheds light on missing variation in worldwide genetic diversity panels
Source: PLoS One. 2018 Oct 26;13(10):e0206512. doi: 10.1371/journal.pone.0206512 (PMC6203392; doi:10.1371/journal.pone.0206512)
Supplement: S1 Fig — The 1,000 Genomes Project sampling is given on a regional basis (top part) or on a population basis (bottom part). In both cases the number of individuals used to evaluate PolyPheMe’s performance is also given (performance test column). (PDF) [file pone.0206512.s001.pdf]

Code	Super population	# Samples (Performance Test)	# Samples (Total)
AFR	African	182	712
AMR	Ad Mixed American	199	373
EAS	East Asian	273	536
EUR	European	338	529
SAS	South Asian	0	543
**TOTAL**		**992**	**2693**

Code	Population	Super population	# Samples (Performance Test)	# Samples (Total)
ACB	African Caribbeans in Barbados	AFR	0	98
ASW	Americans of African Ancestry in SW USA	AFR	52	68
BEB	Bengali from Bangladesh	SAS	0	103
CDX	Chinese Dai in Xishuangbanna, China	EAS	0	108
CEU	Utah Residents (CEPH) with Northern and Western European Ancestry	EUR	53	102
CHB	Han Chinese in Bejing, China	EAS	86	108
CHS	Southern Han Chinese	EAS	97	112
CLM	Colombians from Medellin, Colombia	AMR	70	105
ESN	Esan in Nigeria	AFR	0	111
FIN	Finnish in Finland	EUR	100	105
GBR	British in England and Scotland	EUR	95	102
GIH	Gujarati Indian from Houston, Texas	SAS	0	109
GWD	Gambian in Western Divisions in the	AFR	0	120
IBS	Iberian Population in Spain	EUR	0	108
ITU	Indian Telugu from the UK	SAS	0	112
JPT	Japanese in Tokyo, Japan	EAS	90	105
KHV	Kinh in Ho Chi Minh City, Vietnam	EAS	0	103
LWK	Luhya in Webuye, Kenya	AFR	80	106
MSL	Mende in Sierra Leone	AFR	0	98
MXL	Mexican Ancestry from Los Angeles USA	AMR	59	70
PEL	Peruvians from Lima, Peru	AMR	0	91
PJL	Punjabi from Lahore, Pakistan	SAS	0	108
PUR	Puerto Ricans from Puerto Rico	AMR	70	107
STU	Sri Lankan Tamil from the UK	SAS	0	111
TSI	Toscani in Italia	EUR	90	112
YRI	Yoruba in Ibadan, Nigeria	AFR	50	111
**TOTAL**			**992**	**2693**
