# Supplementary material for: Immune diversity sheds light on missing variation in worldwide genetic diversity panels
Source: PLoS One. 2018 Oct 26;13(10):e0206512. doi: 10.1371/journal.pone.0206512 (PMC6203392; doi:10.1371/journal.pone.0206512)
Supplement: S2 Fig — PolyPheMe’s performance was assessed on a subset of 992 individuals of the 1,000 Genomes Project panel who were already HLA typed using standard methodology. This figure summarizes the concordant/correct, imprecise, and discordant/erroneous results obtained before and after the validation step (S3 Fig). (PDF) [file pone.0206512.s002.pdf]

		Locus					TOTAL	Percent
		HLA-A	HLA-B	HLA-C	HLA-DRB1	HLA-DQB1		
**Before verification**	**Concordant**	1,938	1,944	1,953	1,946	1,359	9,140	97.9
	**Imprecise**	27	16	10	1	1	55	0.6
	**Discordant**	19	24	21	37	36	137	1.5
**After verification**	**Correct**	1,952	1,954	1,974	1,975	1,394	9,249	**99.1**
	**Imprecise**	27	15	8	1	-	51	**0.5**
	**Erroneous**	5	15	2	8	2	32	**0.3**
	**Total**	1,984	1,984	1,984	1,984	1,396	9,332	**100.00**
