# Supplementary material for: Immune diversity sheds light on missing variation in worldwide genetic diversity panels
Source: PLoS One. 2018 Oct 26;13(10):e0206512. doi: 10.1371/journal.pone.0206512 (PMC6203392; doi:10.1371/journal.pone.0206512)
Supplement: S3 Fig — PolyPheMe’s performance was assessed on a subset of 992 individuals of the 1,000 Genomes Project panel who were already HLA typed using standard methodology. 192 HLA types out of 9,332 were initially characterized either as imprecise or discordant comparing to existing results (S2 Fig). For 109 of these 192 cases, we could show that the PolyPheMe results were correct: this figure summarizes the validation steps used for those 109 cases. (PDF) [file pone.0206512.s003.pdf]

Locus	PolyPheMe Result	Existing Result	Valid Result	Nb of cases	Comment
HLA-A	02:07*	02:01*	02:07*	9	One case confirmed by targeted NGS and all cases confirmed manually
	26:02*	26:01*	26:02*	2	Confirmed Manually
	11:02*	11:01*	11:02*	1	Confirmed Manually
	66:03*	66:02*	66:03*	1	Confirmed Manually
HLA-B	27:05*	27:03/51/52/09	27:05*	3	Confirmed Manually
	15:220*	15:03/103	15:220*	2	Confirmed Manually
	40:02*	Homozygous 40:01	40:02*	2	Confirmed Manually
	35:03*	Homozygous 40:01	35:03*	1	Confirmed by targeted NGS and manually
	07:06*	07:05*	07:06*	1	Confirmed Manually
	51:01*	Homozygous 15:01	51:01*	1	Confirmed Manually
	39:24*	39:03	39:24*	1	Confirmed Manually
HLA-C	02:10*	02:02	02:10*	5	One case confirmed by targeted NGS and all cases confirmed manually
	07:18*	07:01	07:18*	4	Three cases confirmed by targeted NGS and all cases confirmed manually
	08:03*	08:01	08:03*	2	Confirmed Manually
	08:22*	Homozygous 08:01	08:22*	2	Confirmed Manually
	18:02*	18:01	18:02*	2	Three cases confirmed by targeted NGS and all cases confirmed manually
	01:03*	01:02	01:03*	1	Confirmed by targeted NGS and manually
	04:01*	Homozygous 03:04	04:01*	1	Confirmed by targeted NGS and manually
	05:37*	05:01/03	05:37*	1	Confirmed by targeted NGS and manually
	06:06*	06:02	06:06*	1	Confirmed Manually
	07:01*	07:02	07:01*	1	Confirmed by targeted NGS and manually
	15:13*	15:02	15:13*	1	Confirmed Manually
HLA-DQB1	02:02*	02:01	02:02*	24	Confirmed Manually
	06:09*	06:05	06:09*	5	Confirmed Manually
	03:19*	03:01	03:19*	4	Confirmed Manually
	03:191*	03:01:01/04/09/19/21/22/24	03:191*	1	Confirmed Manually
	06:39*	06:04/34	06:39*	1	Confirmed Manually
HLA-DRB1	14:54*	14:01	14:54*	11	Confirmed Manually
	04:92*	04:07:01	04:92*	5	Confirmed Manually
	08:03*	08:01/12	08:03*	3	Confirmed Manually
	14:05*	14:01	14:05*	2	Confirmed Manually
	01:23*	01:02	01:23*	1	Confirmed Manually
	08:02*	Homozygous 04:05	08:02*	1	Confirmed Manually
	11:01*	11:04	11:01*	1	Confirmed Manually
	12:01*	Homozygous 13:01	12:01*	1	Confirmed Manually
	12:02*	12:01	12:02*	1	Confirmed Manually
	14:07*	14:44	14:07*	1	Confirmed Manually
	13:02*	13:01	13:02*	1	Confirmed Manually
	13:177*	13:35	13:177*	1	Confirmed Manually
