# Supplementary material for: Immune diversity sheds light on missing variation in worldwide genetic diversity panels
Source: PLoS One. 2018 Oct 26;13(10):e0206512. doi: 10.1371/journal.pone.0206512 (PMC6203392; doi:10.1371/journal.pone.0206512)
Supplement: S4 Fig — This figure summarizes the total number of in silico HLA types realized by PolyPheMe on a locus-by-locus basis. For each locus, the number of precise and imprecise types is given, together with the number of cases for which no type was obtained. (PDF) [file pone.0206512.s004.pdf]

Locus	# types	Precise types	Imprecise types	No result
**HLA-A**	5,386	5,346	44	4
**HLA-B**	5,386	5,377	25	16
**HLA-C**	5,386	5,372	18	4
**HLA-DQB1**	4,798	4,760	41	3
**HLA-DRB1**	5,386	5,387	4	5
**TOTAL**	**26,342**	**26,178**	**132**	**32**
