# Supplementary figures and images for: Immune diversity sheds light on missing variation in worldwide genetic diversity panels
Source: PLoS One. 2018 Oct 26;13(10):e0206512. doi: 10.1371/journal.pone.0206512 (PMC6203392; doi:10.1371/journal.pone.0206512)

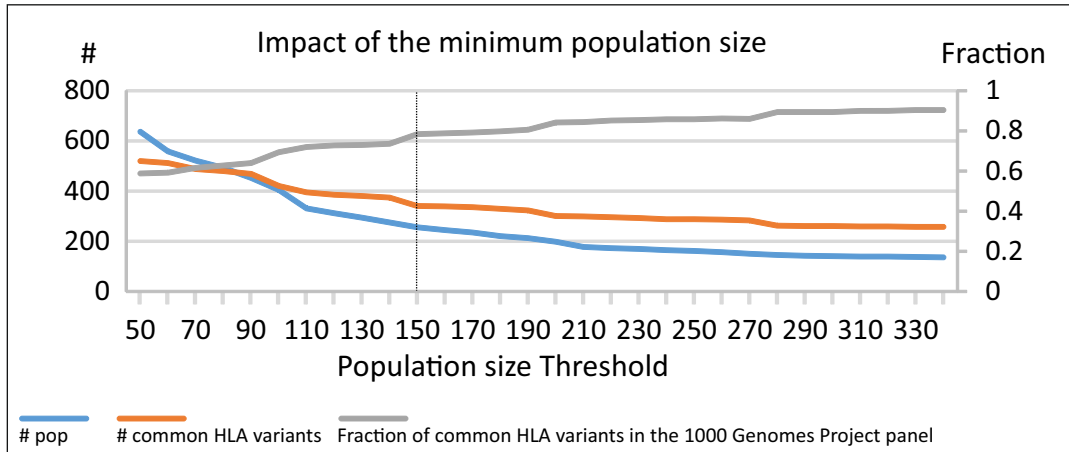

Supplement: S5 Fig — A threshold of at least 150 individuals per population was used for the final analysis (dotted line). (PDF) [file pone.0206512.s005.pdf]
