# Supplementary material for: Immune diversity sheds light on missing variation in worldwide genetic diversity panels
Source: PLoS One. 2018 Oct 26;13(10):e0206512. doi: 10.1371/journal.pone.0206512 (PMC6203392; doi:10.1371/journal.pone.0206512)
Supplement: S6 Fig — For each occurrence threshold (minimum number of observations of a given allele in populations), the number of alleles of HLA-A, -B, -C,–DQB1, and–DRB1 observed with this minimum occurrence is given (line ‘1.’). The second line (‘2.’) indicates how many of the alleles listed in 1. are also observed in the 1,000 Genomes Project panel. Finally the third line corresponds to the fraction ‘2./1’. For example, 1,202 HLA-A, -B, -C,–DQB1, and–DRB1 alleles are observed at least five times in populations. Out of those 1,202 alleles, 354 are also observed in the 1,000 Genomes Project panel, which corresponds to a fraction of 0.29. (PDF) [file pone.0206512.s006.pdf]

	# occurrences minimum											
	1	2	3	4	5	6	7	8	9	10	100	1000
1. # HLA alleles (total)	2935	1993	1581	1354	1202	1105	1027	970	925	888	455	280
2. # HLA alleles (given in line 1 and also observed in the 1,000 Genomes Project)	385	376	364	359	354	351	349	348	348	345	313	260
Ratio (# in line 2 divided by # in line 1)	0.13	0.19	0.23	0.27	0.29	0.32	0.34	0.36	0.38	0.39	0.69	0.93
