# Supplementary material for: Immune diversity sheds light on missing variation in worldwide genetic diversity panels
Source: PLoS One. 2018 Oct 26;13(10):e0206512. doi: 10.1371/journal.pone.0206512 (PMC6203392; doi:10.1371/journal.pone.0206512)
Supplement: S7 Fig — This figure summarizes the common HLA haplotypes and the populations that harbor them with frequency > 1%. The third column gives the number of observations of the haplotypes in the 1,000 Genomes Project panel. Population codes correspond to those described in S8 Fig. Two haplotypes that are not observed in the 1,000 Genomes Project are in bold because they carry common HLA variants (HLA-C*08:06 and HLA-DRB1*14:08). (PDF) [file pone.0206512.s007.pdf]

Haplotype	Populations with frequency > 0.01	# Observations
A*01:01, B*57:01, C*06:02, DRB1*07:01, DQB1*03:03	AINDI, SCSEAI	50
A*01:01, B*08:01, C*07:01, DRB1*03:01, DQB1*02:01	AAFA, AFA, AISC, ALANAM, AMIND, CARHIS, CARIBI, CAU, EURCAU, HAWI, HIS, MENAFC, MSWHIS, NAM, SCAHIS, SCAMB	49
A*03:01, B*07:02, C*07:02, DRB1*15:01, DQB1*06:02	AISC, ALANAM, AMIND, CARHIS, CAU, EURCAU, HIS, MENAFC, MSWHIS, NAM, SCAHIS	33
A*29:02, B*44:03, C*16:01, DRB1*07:01, DQB1*02:01	AISC, AMIND, CARHIS, CARIBI, CAU, EURCAU, HIS, MSWHIS, NAM, SCAHIS	30
A*03:01, B*35:01, C*04:01, DRB1*01:01, DQB1*05:01	CAU, EURCAU	30
A*33:03, B*58:01, C*03:02, DRB1*03:01, DQB1*02:01	AINDI, API, FILII, KORI, NCHI, SCSEAI, VIET	29
A*11:01, B*15:02, C*08:01, DRB1*12:02, DQB1*03:01	API, NCHI, VIET	29
A*33:03, B*44:03, C*07:01, DRB1*07:01, DQB1*02:01	AINDI, API, KORI, SCSEAI, VIET	24
A*02:07, B*46:01, C*01:02, DRB1*09:01, DQB1*03:03	API, NCHI, VIET	24
A*26:01, B*08:01, C*07:02, DRB1*03:01, DQB1*02:01	AINDI, SCSEAI	23
A*30:01, B*42:01, C*17:01, DRB1*03:02, DQB1*04:02	AAFA, AFA, AFB, CARB, SCAMB	20
A*24:02, B*52:01, C*12:02, DRB1*15:02, DQB1*06:01	JAPI, KORI	20
A*02:01, B*44:02, C*05:01, DRB1*04:01, DQB1*03:01	AISC, ALANAM, AMIND, CAU, EURCAU, NAM	17
A*01:01, B*37:01, C*06:02, DRB1*10:01, DQB1*05:01	AINDI, SCSEAI	17
A*33:03, B*58:01, C*03:02, DRB1*13:02, DQB1*06:09	KORI, NCHI, VIET	14
A*24:02, B*35:02, C*04:01, DRB1*11:04, DQB1*03:01	MENAFC	14
A*02:01, B*07:02, C*07:02, DRB1*15:01, DQB1*06:02	AISC, ALANAM, AMIND, CAU, EURCAU, NAM	13
A*29:01, B*07:05, C*15:05, DRB1*10:01, DQB1*05:01	VIET	13
A*30:01, B*13:02, C*06:02, DRB1*07:01, DQB1*02:01	API, KORI, NCHI	11
A*33:03, B*44:03, C*14:03, DRB1*13:02, DQB1*06:04	JAPI, KORI	9
A*24:02, B*07:02, C*07:02, DRB1*01:01, DQB1*05:01	JAPI, KORI	7
A*24:02, B*54:01, C*01:02, DRB1*04:05, DQB1*04:01	JAPI, KORI	7
A*02:07, B*46:01, C*01:02, DRB1*08:03, DQB1*06:01	JAPI, KORI	7
A*24:02, B*39:06, C*07:02, DRB1*14:06, DQB1*03:01	MSWHIS	5
A*33:01, B*14:02, C*08:02, DRB1*01:02, DQB1*05:01	MENAFC	4
A*68:02, B*57:03, C*07:01, DRB1*03:02, DQB1*04:02	CARHIS, CARIBI	4
A*11:01, B*46:01, C*01:02, DRB1*09:01, DQB1*03:03	NCHI	4
A*24:07, B*35:05, C*04:01, DRB1*12:02, DQB1*03:01	FILII, HAWI, VIET	3
A*24:02, B*15:02, C*08:01, DRB1*12:02, DQB1*03:01	VIET	3
A*11:01, B*54:01, C*01:02, DRB1*04:05, DQB1*04:01	JAPI	3
A*11:01, B*15:01, C*04:01, DRB1*04:06, DQB1*03:02	JAPI, KORI	3
A*68:03, B*39:05, C*07:02, DRB1*04:07, DQB1*03:02	MSWHIS	1
A*11:01, B*13:01, C*03:04, DRB1*15:01, DQB1*06:01	NCHI	1
A*11:01, B*38:02, C*07:02, DRB1*15:02, DQB1*05:02	FILII	1
A*24:02, B*59:01, C*01:02, DRB1*04:05, DQB1*04:01	JAPI	1
A*24:02, B*38:02, C*07:02, DRB1*15:02, DQB1*05:02	FILII	1
A*24:02, B*40:01, C*04:01, DRB1*04:03, DQB1*03:02	FILII, HAWI	1
A*02:01, B*40:01, C*03:04, DRB1*09:01, DQB1*03:03	HAWI	0
A*02:03, B*15:02, C*08:01, DRB1*12:02, DQB1*03:01	VIET	0
A*02:06, B*15:01, C*01:02, DRB1*14:02, DQB1*03:01	ALANAM	0
A*02:06, B*27:05, C*02:02, DRB1*14:02, DQB1*03:01	ALANAM	0
A*02:06, B*40:02, C*03:04, DRB1*14:02, DQB1*03:01	ALANAM	0
A*02:06, B*55:02, C*01:02, DRB1*12:01, DQB1*03:01	HAWI	0
A*11:01, B*15:35, C*07:02, DRB1*15:02, DQB1*05:02	FILII	0
A*11:01, B*48:01, C*08:01, DRB1*11:01, DQB1*03:01	HAWI	0
A*24:02, B*27:05, C*02:02, DRB1*14:02, DQB1*03:01	ALANAM	0
A*24:02, B*40:02, C*03:04, DRB1*04:01, DQB1*03:01	ALANAM	0
A*24:02, B*40:02, C*03:04, DRB1*14:01, DQB1*05:03	ALANAM	0
A*24:02, B*40:02, C*03:04, DRB1*14:02, DQB1*03:01	ALANAM	0
A*24:02, B*40:02, C*15:02, DRB1*04:05, DQB1*05:03	FILII	0
A*24:02, B*48:01, C*08:01, DRB1*08:03, DQB1*06:01	HAWI	0
A*24:02, B*48:01, C*08:01, DRB1*14:02, DQB1*03:01	ALANAM	0
**A*24:02, B*48:01, C*08:06, DRB1*04:01, DQB1*03:01**	ALANAM	0
A*24:03, B*51:06, C*12:04, DRB1*07:01, DQB1*03:03	FILII	0
A*34:01, B*15:21, C*04:03, DRB1*15:02, DQB1*05:02	FILII	0
A*34:01, B*38:02, C*07:02, DRB1*15:02, DQB1*05:02	FILII	0
**A*34:01, B*40:02, C*15:02, DRB1*14:08, DQB1*05:03**	HAWI	0
A*34:01, B*40:02, C*15:02, DRB1*15:02, DQB1*05:02	FILII	0
