# Supplementary material for: Immune diversity sheds light on missing variation in worldwide genetic diversity panels
Source: PLoS One. 2018 Oct 26;13(10):e0206512. doi: 10.1371/journal.pone.0206512 (PMC6203392; doi:10.1371/journal.pone.0206512)
Supplement: S8 Fig — This figure provides the population codes used in the haplotype analysis (S7 Fig). (PDF) [file pone.0206512.s008.pdf]

Code	Population
AAFA	African American
AFA	African American
AFB	African
AINDI	South Asian Indian
AISC	American Indian - South or Central Am.
ALANAM	Alaska Native or Aleut
AMIND	North American Indian
API	Asian or Pacific Islander
CARB	Caribbean Black
CARHIS	Caribbean Hispanic
CARIBI	Caribbean Indian
CAU	Caucasian
EURCAU	European Caucasian
FILII	Filipino API
HAWI	Hawaiian or other Pacific Islander
HIS	Hispanic
JAPI	Japanese
KORI	Korean
MENAFC	Middle Eastern or N. Coast of Africa
MSWHIS	Mexican or Chicano
NAM	Native American
NCHI	Chinese
SCAHIS	Hispanic - South or Central American
SCAMB	Black - South or Central American
SCSEAI	Southeast Asian
VIET	Vietnamese
