# Supplementary material for: Immune diversity sheds light on missing variation in worldwide genetic diversity panels
Source: PLoS One. 2018 Oct 26;13(10):e0206512. doi: 10.1371/journal.pone.0206512 (PMC6203392; doi:10.1371/journal.pone.0206512)
Supplement: S1 File — A description of PolyPheMe software is provided: general description, typing strategy, typing steps, results, and computing power requirements and availability. (PDF) [file pone.0206512.s013.pdf]

PolyPheMe HLA typing tool

General description

The HLA version of PolyPheMe software (Xegen, France) is a novel *in silico* solution to perform highly accurate HLA typing (*HLA-A*, *-B*, *-C*, *-DRB1*, and *-DQB1* in this study) from a wide range of sequence data, including targeted, whole exome, or whole genome sequencing. Depending on the type of sequence data used, PolyPheMe provides different levels of typing resolution: for example, allotype-level typing if exon sequences are used (Field 2 or 3 in the HLA nomenclature [1]) or full allele-level typing if exon and intron sequences (whole genome or targeted approaches) are used (Field 4 in the HLA nomenclature [1]). All analyses in this study were performed with PolyPheMe v1.2 on exome sequences using the IMGT database 3.28 as reference [2].

Typing strategy

Unlike HLA typing methods that rely on statistics, PolyPheMe is based on qualitative approaches. In particular, for many of the steps in the analysis, the software only works with sequence reads that are specific of the locus and allele investigated. As a result of this design, it is possible to perform accurate HLA typing even with low coverage data or with exome data that is heterogeneous in terms of coverage.

Typing steps

Stage 1

When the HLA typing is performed from genome-wide sequence data, a first step in the analysis is to isolate all the reads related to the HLA loci investigated so that subsequent steps are only performed on those isolated reads, hence dramatically reducing the time required for data analysis. This step uses Bowtie 2 [3] and typically precedes the use of PolyPheMe.

Stage 2

The reads isolated in the first step are then assigned to one of the loci analyzed using an “end to end” mapping step with Bowtie 2 [3]. This step allows retrieval of highly specific reads for all loci based on the use of positive and negative references. The reference datasets rely on population genetics data and only include the HLA alleles of each locus

investigated when they have frequencies >0.05 in at least one population (as defined in Allele Frequency Net Database [4]). The negative reference dataset includes all the alleles from related loci (HLA gene and pseudogene sequences). At the end of this stage, the software generates a fastq file for each locus that contains only the sequence reads specific of that locus.

Stage 3

For each locus, the software will then determine “allele group”-level types (Field 1 in the HLA nomenclature [1]). To do this, locus-specific reads are mapped against each of the possible allele groups (each group being represented by a selection of the most common alleles of this group, as defined in Allele Frequency Net Database [4]) to define which groups have the largest number of locus-specific reads. This step is a multipass process where the least-represented group is eliminated at each pass. When only two allele groups are left, a threshold of 12% is used to define whether the input individual sample is homozygous or heterozygous (i.e. when less than 12% of the reads are associated with one allele group, this group is eliminated). This step allows to define one (homozygous individual) or two (heterozygous individual) allele groups for each locus.

Stage 4

Once allele groups are defined for each locus, the final stage of the analysis is to increase the resolution of the typing to at least reach “specific HLA protein”-level typing (Field 2 in the HLA nomenclature [1]). Depending on the number of allele groups identified in stage 3, one or two alleles can be identified for each allele group. Allele identification is first made by analyzing exons and then, if available, introns. In this analysis, all the possible variable positions within the allele group are studied one by one to determine if they are heterozygous or homozygous, using a dynamic threshold dependent on the coverage and on the heterozygous character of the locus (one or two allele groups identified). Using IMGT data as reference [2], known alleles that display discordant sequences comparing to the sequence reads of the individual investigated are eliminated from the list of possible types. For loci with two distinct alleles within the same allele group, a phasing step is performed to identify the two alleles: this step uses a custom algorithm that considers data from the reads, read pairs and overlap between reads with allele-specific positions. At the end of this stage, a typing solution is provided.

Results

At the end of the analysis, PolyPheMe typically provides one (homozygous) or two (heterozygous) types per locus, as well as the sequence reads associated with each type. Solutions can sometimes be ambiguous when the sequence data are insufficient at variable positions (low coverage). A solution is defined as ambiguous when the final result includes three or more possible alleles or when more than one allele is predicted but no heterozygous position was detected and only one allele group is identified. In such cases, a CWD filter (Version 2.0.0) [5] can be applied to eliminate "rare alleles or not validated alleles" and only list those defined as common.

Computing power requirements and availability

Developed in Java, PolyPheMe can be used on a wide range of operating systems (Windows, Linux, etc...). The software can be run on a desktop computer with limited resources i.e. all analyses in this study were for example produced on a computer with a quadri-core processor and >4GB of RAM. Disk space requirement varies according to the type of sequence data used. Academic or commercial licenses of PolyPheMe can be purchased by contacting XEGEN company (direction@xegen.fr).

References

1. Marsh SG, Albert ED, Bodmer WF, Bontrop RE, Dupont B, Erlich HA, et al. Nomenclature for factors of the HLA system, 2010. Tissue Antigens. 2010;75(4):291-455. doi: 10.1111/j.1399-0039.2010.01466.x. PubMed PMID: 20356336; PubMed Central PMCID: PMC2848993.
2. Robinson J, Halliwell JA, Hayhurst JD, Flicek P, Parham P, Marsh SG. The IPD and IMGT/HLA database: allele variant databases. Nucleic acids research. 2015;43(Database issue):D423-31. doi: 10.1093/nar/gku1161. PubMed PMID: 25414341; PubMed Central PMCID: PMC4383959.
3. Langmead B, Salzberg SL. Fast gapped-read alignment with Bowtie 2. Nat Methods. 2012;9(4):357-9. doi: 10.1038/nmeth.1923. PubMed PMID: 22388286; PubMed Central PMCID: PMC3322381.
4. Gonzalez-Galarza FF, Christmas S, Middleton D, Jones AR. Allele frequency net: a database and online repository for immune gene frequencies in worldwide populations. Nucleic acids research. 2011;39(Database issue):D913-9. doi: 10.1093/nar/gkq1128. PubMed PMID: 21062830; PubMed Central PMCID: PMC3013710.
5. Mack SJ, Cano P, Hollenbach JA, He J, Hurley CK, Middleton D, et al. Common and well-documented HLA alleles: 2012 update to the CWD catalogue. Tissue Antigens. 2013;81(4):194-203. doi: 10.1111/tan.12093. PubMed PMID: 23510415; PubMed Central PMCID: PMC3634360.
